# Evaluating Phylogenetic Informativeness as a Predictor of Phylogenetic Signal for Metazoan, Fungal, and Mammalian Phylogenomic Data Sets

**DOI:** 10.1155/2013/621604

**Published:** 2013-06-26

**Authors:** Francesc López-Giráldez, Andrew H. Moeller, Jeffrey P. Townsend

**Affiliations:** ^1^Department of Ecology and Evolutionary Biology, Yale University, 165 Prospect Street, New Haven, CT 06520, USA; ^2^Department of Biostatistics, Yale University, 135 College Street, New Haven, CT 06520, USA; ^3^Program in Computational Biology and Bioinformatics, 300 George Street, Yale University, New Haven, CT 06520, USA

## Abstract

Phylogenetic research is often stymied by selection of a marker that leads to poor phylogenetic resolution despite considerable cost and effort. Profiles of phylogenetic informativeness provide a quantitative measure for prioritizing gene sampling to resolve branching order in a particular epoch. To evaluate the utility of these profiles, we analyzed phylogenomic data sets from metazoans, fungi, and mammals, thus encompassing diverse time scales and taxonomic groups. We also evaluated the utility of profiles created based on simulated data sets. We found that genes selected via their informativeness dramatically outperformed haphazard sampling of markers. Furthermore, our analyses demonstrate that the original phylogenetic informativeness method can be extended to trees with more than four taxa. Thus, although the method currently predicts phylogenetic signal without specifically accounting for the misleading effects of stochastic noise, it is robust to the effects of homoplasy. The phylogenetic informativeness rankings obtained will allow other researchers to select advantageous genes for future studies within these clades, maximizing return on effort and investment. Genes identified might also yield efficient experimental designs for phylogenetic inference for many sister clades and outgroup taxa that are closely related to the diverse groups of organisms analyzed.

## 1. Introduction

The genomes of nearly 400 eukaryotes and nearly 3000 prokaryotes are now or are in the process of being sequenced. Most of these organisms have thousands of genes, yet only a few of those have been commonly used as markers for phylogenetic studies [1]. In cases where choice has been exercised, genes have been selected for sequencing based on rough impressions of the genes' utilities in previous studies of taxa that are to varying degrees divergent from the taxa of interest. Recent sequencing of multiple genomes within major branches of the tree of life provides a much greater selection of markers and excites hope that more accurate procedures for experimental design may be adopted. However, despite the phenomenal growth in sequence information available, the optimal way to employ genome-wide data sets to inform more clade-specific molecular phylogenetic studies remains elusive [2, 3] due to a lack of methods that quantitatively assess the power of genes to resolve particular nodes in a phylogeny. 

Although a few rules of thumb for selecting genes for phylogenetic studies have been advocated (e.g., percent sequence divergence; 4, 5; or proportion of parsimony-informative sites; 6), their successful use is highly context dependent. Conventional wisdom dictates selection of a gene that evolves at an appropriate pace for the phylogenetic question of interest, but this axiom often fails to illuminate the correct decision. Fairly complex distributions of rates across characters can yield information regarding some periods of the history encompassed by the phylogeny but not others [4–6].

 In response to the crucial role of gene selection in experimental design [4, 7–11], Townsend [5] proposed a metric that predicts utility across historical time for known genes. Based on the estimated full distribution of rates across characters, the Townsend [5] informativeness yields a graphical appraisal of a gene's signal for any historical epoch. To estimate informativeness, prior data on the molecular evolutionary rates for each site of a locus is required. This prior information may be derived from three potential sources: (1) preliminary data on the candidate loci from a well-studied subset of the taxa of interest; (2) data on the candidate loci from a well-studied sister clade; or (3) comparative genomic data from sequenced genomes within and/or outside the clade of interest. Thus, Townsend [5] informativeness metrics can be obtained without reference to sequence data from the taxa of interest.

To evaluate phylogenetic informativeness as a procedure for selecting loci to sequence for phylogenetic studies that incorporate broad taxon sampling, we utilized empirical data sets for which the process of evolution may only be approximated, as well as simulated data for which we could specify the process and the true tree. In each case, we tested the performance of the Townsend [5] phylogenetic informativeness with trees with more than four taxa and its robustness to the effects of homoplasy. We analyzed data sets encompassing different time scales and taxonomic groups: (i) mammal data sets [12] consisting of 50 genes each (~33,440 aa and ~100,649 bp) sequenced in 20 species; (ii) a fungal data set [13] consisting of 46 genes (~13,082 aa) sequenced in 28 species; (iii) an animal/fungal data set [14, 15] consisting of 50 genes (~12,089 aa) sequenced in 25 species. In parallel, we simulated 50 amino acid and 50 DNA sequence alignments of 300 sites each. Genes for empirical and simulated data sets were ranked by phylogenetic informativeness and analyzed for their ability to recapitulate known node identity and robustness using measures of branch support associated with maximum likelihood and maximum parsimony optimality criteria. Genes from the empirical data sets examined here that were identified as performing well could be especially useful for phylogenetic inference in organisms related to the clades analyzed. Our results represent the first phylogenomic test of the phylogenetic informativeness method presented by Townsend [5], supporting it as a metric of potential phylogenetic signal in both nucleotide and amino acid data sets.

## 2. Materials and Methods 

### 2.1. Sequence Data Composition

**Figure 1:**
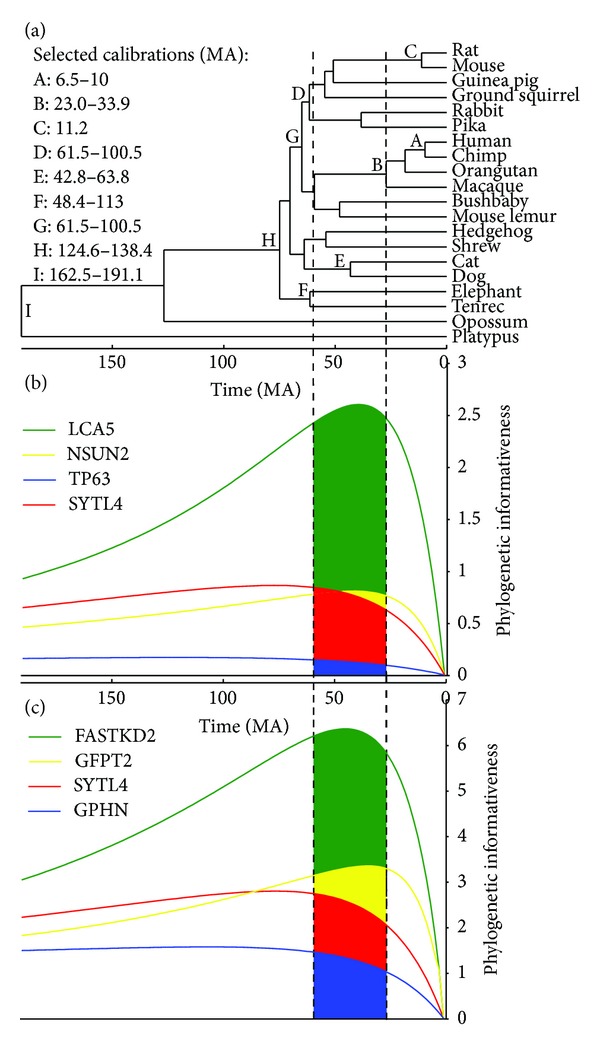
Phylogenetic informativeness profiles for the OrthoMaM data set. (a) Chronogram and the calibration points used to calculate site rates. Phylogenetic informativeness profiles over a 190 Myr period for (b) four amino acid sequence alignments and (c) four DNA sequence alignments, on the same time scale as in panel (a). Integration of the area below the profiles can provide a ranking of the predicted utility of genes for that epoch (here, the epoch encompassing the branch leading to primates). Integration results will be the largest for the genes that have the highest probability of exhibiting mutations during the given epoch that will not be obscured in subsequent branches. To quantitatively establish genes that will be most informative for the entire phylogeny, integrals over the whole time scale were calculated.

**Figure 2:**
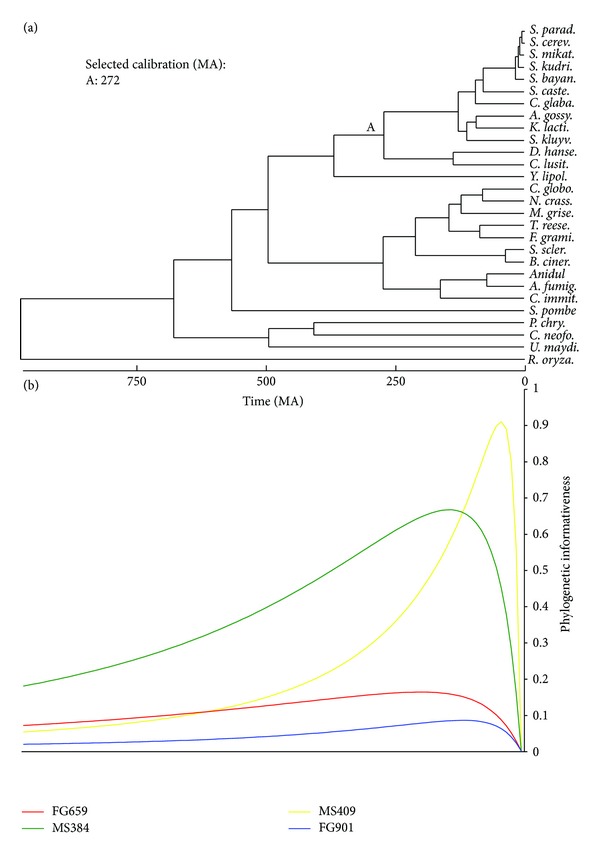
Phylogenetic informativeness profiles for the FUNYBASE data set. (a) Chronogram and the calibration points used to calculate site rates. (b) Phylogenetic informativeness profiles over a 973 Myr period for four amino acid sequence alignments, on the same time scale as in panel (a).

**Figure 3:**
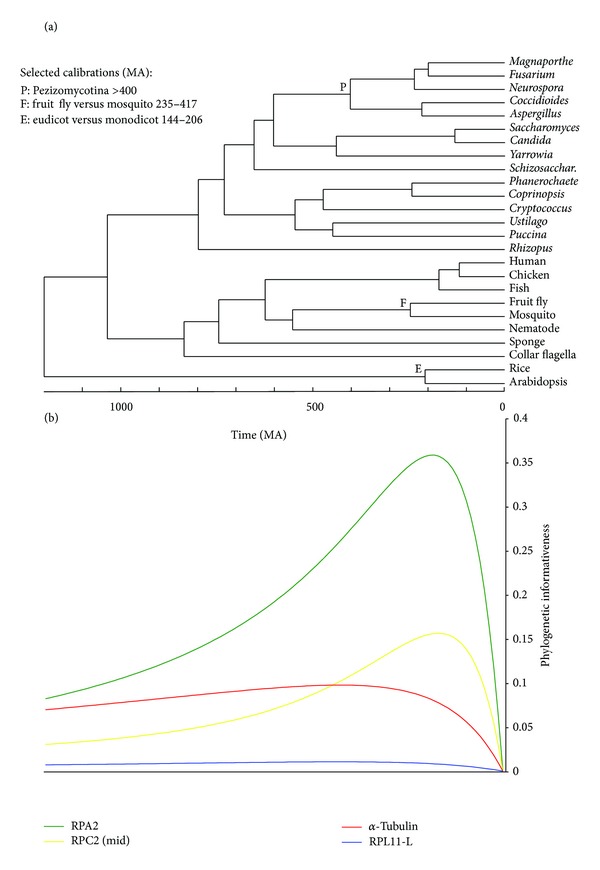
Phylogenetic informativeness profiles for the TBR data set. (a) Chronogram and the calibration points used to calculate site rates. (b) Phylogenetic informativeness profiles over a 1193 Myr period for four amino acid sequence alignments, on the same time scale as in panel (a).

We obtained four amino acid and two DNA sequence data sets for analysis. In all cases, species were selected whose phylogeny has been well established. Data sets encompassed different time scales and taxonomic levels, and were extracted from four different sources (Table 1). From the OrthoMaM database [12] of orthologous genomic markers for placental mammals, we obtained a data set of amino acid and a data set of nucleotide sequences. We selected 50 single-copy orthologous genes that were present in 20 species (Figure 1(a)) and that had lengths of ~2000 bp and ~666 aa (see Supplementary Tables 1 and 2 in Supplementary Material available online at http://dx.doi.org/10.1155/2013/621604). The large number of genes in OrthoMaM facilitated a gene size constraint to minimize the influence of sequence length in the phylogenetic inference. Forty genes were in common between the amino acid and DNA data sets. Northern tree shrew, cow, horse, little brown bat, and nine-banded armadillo species were also present in OrthoMaM database but were excluded from the analyses to ensure a comparison of outcomes against a well-resolved and uncontroversial phylogenetic tree. From the FUNYBASE database [13] of fungal orthologous sequences, we obtained amino acid sequences for 46 genes (Supplementary Table 3) in 28 fungal species (Figure 2(a)). *Stagonospora nodorum* and *Aspergillus oryzae* were also present in FUNYBASE but they were excluded due to their weakly supported phylogenetic placement [16]. The third source was Taylor and Berbee [15], modified from Rokas et al. [14]—abbreviated as the TBR data set—from which we obtained an alignment of 50 amino acid sequence regions (Supplementary Table 4) from 8 animal, 15 fungal, and 2 plant species (Figure 3(a)). 

The two sets of simulated alignments, amino acid and nucleotide, were generated with Seq-Gen v1.3.2 software [17]. Simulated alignments allowed us to fix the alignment length, the evolutionary model, and its parameters. To ensure that the simulations encompassed a realistic instance, the parameter values for mean site rate, proportion of invariant sites, and sequence length approximated the values found in the FUNYBASE data set. Also, both amino acid and DNA sequences were simulated on the FUNYBASE chronogram (see below). The gamma rate heterogeneity values were varied to explore a wide range. JTT and K2P (with *κ* = 2) models were used for amino acid and nucleotide alignments, respectively. For both amino acid and DNA, 50 different data sets were simulated, each based on one of 10 different mean rates ranging from 0.0001 to 0.001 substitutions per site per Myr and ranging across five gamma rate heterogeneity values, including no rate heterogeneity and *α* = 0.5, 1, 2, and 3. In all cases, the gamma distribution was discretized into 10 rate categories. For every alignment, 20% of sites were set to be invariant. For each of these 50 alignments, we generated 10 replicates. The Seq-Gen program assigns each site to either the invariant category or one of the gamma categories stochastically within each simulation. As a result, the number of invariant sites and the number of sites in each category vary from simulation to simulation. However, this fact would not guarantee that each rate category has the same number of sites. To ensure that each replicated alignment had the same exact rate distribution but not the same amino acid or DNA sequence, replicates were created such that each replicate contained 60 invariant sites and 24 sites for each of the 10 rate categories if rate heterogeneity was specified. 

The sequences downloaded from OrthoMaM and FUNYBASE were aligned using MUSCLE v3.6 [18] with default settings. Gblocks v0.91b [19] was used to remove ambiguously aligned positions from the alignments. In Gblocks, the minimum number of sequences for a flank position was set to 16. Only sites with more than half of sequences with gaps were treated as a gap position and eliminated from the final alignment. Default settings were applied for the rest of options.

### 2.2. Divergence Times and Chronogram

To compute the rates of evolution of amino acid and nucleotide sites for all nonsimulated data sets, we specified an ultrametric evolutionary tree. The concatenated amino acid sequences were used in each case to estimate the phylogeny with the parallel version of MrBayes v3.1.2 [20, 21]. The length of the concatenated sequences totaled 16,802, 13,082, and 12,089 aa for OrthoMaM, FUNYBASE, and TBR alignments, respectively. We allowed mixed models with invariant sites and gamma-shaped rate variation with four rate categories. All parameters were unlinked; thus, the models and parameters were estimated during the analysis separately for each locus. Ten independent runs were conducted using 4 MCMC chains and random starting trees of 500,000 generations each, sampling trees every 100 generations. We discarded the first 100,000 generations as burn-in after visualization in the program Tracer v1.4 [22], long after the log likelihood reached apparent stationarity. 

For convenience, we used a time-calibrated phylogeny (chronogram). While absolute dates of internal nodes were not relevant to any inferences herein, their relative depths were aligned with the ultrametric profiles for predictive purposes. We obtained the chronogram for each data set (see Figures 1(a)–3(a)) by passing the phylogenetic tree with the highest likelihood to r8s software v1.71 [23]. This software allows incorporation of multiple calibration points, fixing or constraining minimal or maximal ages to the nodes. For the OrthoMaM chronogram (Figure 1(a)), we used diverse calibration points from [24]. For the FUNYBASE chronogram (Figure 2(a)), the tree was calibrated by fixing the split of *D. hansenii* and *C. albicans* from the other yeasts at 272 Myr [25]. For the TBR chronogram (Figure 3(a)), three calibration points were used as in the intermediate solution in [15]. Divergence times were estimated by the penalized likelihood method with a truncated Newton algorithm in r8s, setting the smoothing parameter to 0.06 for OrthoMaM and 0.01 for FUNYBASE trees. The optimization of the smoothing parameter was obtained using the cross-validation feature in r8s following the instructions of the program manual (available at http://loco.biosci.arizona.edu/r8s/). As indicated in [15], there was an absence of predictable lineage-specific rate correlations in the TBR tree. Thus, these data were processed following the recommendation of the r8s program documentation, with the Langley-Fitch method that assumes a global substitution rate instead of the penalized likelihood method.

### 2.3. Evolutionary Rates and Phylogenetic Informativeness

Using the alignment data and its corresponding chronogram, molecular evolutionary rates were estimated for each gene at each alignment position. We used Rate4site [26] and DNArates (Olsen, unpublished) programs to obtain the substitution rates at amino acid and nucleotide sites, respectively. These programs were chosen because they provide ML approach to estimate the rates for each site independently and a simple model to avoid overparameterization. In the Rate4site program, rates were inferred assuming a JTT model for the topology and branch lengths of the input phylogenetic tree without any optimization. In the DNArates program, rates were inferred assuming K2P (with *κ* = 2) model. 

**Figure 4:**
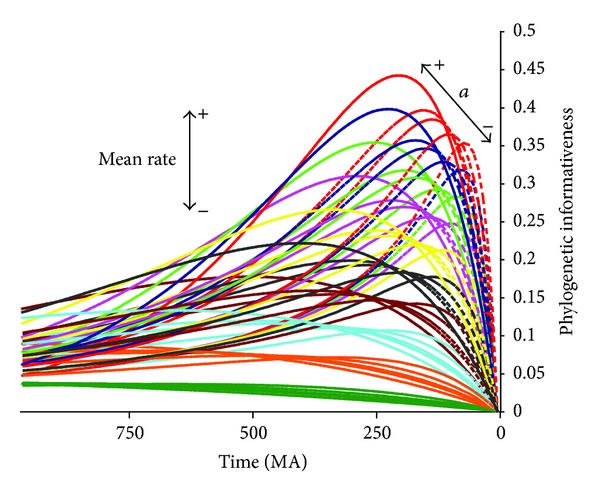
Phylogenetic informativeness profiles for simulated amino acid and DNA alignments, on the same time scale as in Figure 1 (973 Myr period). Each of the 10 different colors represents a different mean rate, from 0.0001 (slowest, bottom) to 0.001 (fastest, top) substitutions per site per million years. Dashed lines are profiles from alignments simulated with gamma rate heterogeneity (*α* = 0.5, 1, 2, and 3).

For each gene, the phylogenetic informativeness profile *ρ* as a function of time, *T*, was calculated as

(1) $$\begin{array}{c} \;\rho \left(T;\lambda_{1},\ldots ,\lambda_{2}\right)=\sum_{i=1}^{n}16\lambda_{i}^{2}Te^{-4{T\lambda }_{i}}, \end{array}$$

substituting the estimated rates *λ*_*i*_ of evolution of each site [5]. This formula provides the probability that character *i* would provide an unambiguous synapomorphy lying within an asymptotically short internode between two pairs of sister taxa whose common ancestor is at time *T*. To convey the informativeness of a particular data set, the equation was plotted at a continuum of depths, from time 0 to the root of the phylogenetic trees (Figures 1–4). The differential phylogenetic informativeness (DPI) of each gene was evaluated quantitatively by integrating over the phylogenetic informativeness profile from the origin (*h*_1_) to the terminus (*h*) of the epochs of interest, ∫_*h*1_^*h*2^ *ρ*(*T*; *λ*)*T*. Assigning *h*_1_ and *h*_2_ so as to encompass all branching points of a phylogeny provided a summary of the relative informativeness of each gene to resolve all nodes in the phylogeny. Using DPI, we ranked the genes for each data set. 

Both the calculations of the molecular evolutionary rate and of the phylogenetic informativeness profiles were performed using the PhyDesign web application [27].

### 2.4. Phylogenetic Analysis

To evaluate the performance of each locus, we analyzed the accuracy and robustness with which each locus recovered the well-established topology. In each case, the phylogeny resulting from our analysis of the concatenated amino acid alignments exactly matched established topology as described in the literature [12, 16, 28, 29]. To assess the fidelity with which individual genes recovered the reference tree on a holistic scale, we calculated the topological distance between each individual gene tree and the concatenated tree using the Symmetric Difference [30] computed by the TreeDist program included in the Phylip v3.68 package [31]. To measure the robustness with which each gene recovered the correct topology on a node-by-node basis, we applied four metrics of branch support based on two different optimization criteria, maximum likelihood (ML) and maximum parsimony (MP). The ML support metrics were nonparametric bootstrap (ML-BP) and the approximate Likelihood-Ratio Test statistic (aLRT statistic; [32]). The MP support metrics were nonparametric bootstrap (MP-BP) and Decay Index (DI) [33]. 

For all individual aligned orthologous markers, we determined the amino acid and nucleotide substitution models that best fit the data using the command-line mode of ProtTest v1.4 [34] and ModelGenerator v0.85 [35], respectively. In both programs, the models for each gene were selected to minimize the BIC criterion. 

We inferred ML gene trees with PHYML v2.4.4 [36] using bootstrap proportions (BP) based on 100 bootstrap replicates, with the model and parameters as described above. For empirical data sets, we discretized the gamma site-rate distribution into four rate categories. For simulated sequences, we discretized the gamma site-rate distribution into 10 categories, both for inference and for simulation. Ten instead of four rate categories were used in the simulated data to obtain more diverse sequences and substitution rates during the simulation process. We used PAUP* v4.0b10 [37] for MP-BP analyses based on 1000 BP replicates. A heuristic search with TBR branch-swapping on 20 random sequence addition replicate starting trees was employed. 

Although BP values are probably the most frequently used type of support values, they scale nonlinearly with the number of synapomorphies, conveying little information when they are low and reaching an asymptote of 100 rapidly when they are high. In contrast, aLRT and DI are not constrained by an upper limit. The aLRT is based on the conventional LRT under the null hypothesis that the inferred branch has length 0 [32]. Our analysis, applied the aLRT statistic value—that is, two times the difference between the maximum log-likelihood values of the best and the second best alternative arrangements around the branch of interest—with the modified version of PHYML v2.4.5 [32]. The last support measure, DI, also known as Bremer support, is the number of parsimony steps from the best tree to the next best tree without the branch of interest. DIs were calculated with the help of AutoDecay v5.04 for PERL using reverse constraints in PAUP*. 

### 2.5. Statistical Analysis

We used three different statistical approaches to evaluate the performance of phylogenetic informativeness. First, we correlated the DPI gene ranking with the tree distance from the gene tree to the well-established tree topology. We expected that the genes ranked highest would show a low tree distance—that is, recovering a topology closest to the reference tree. Second, we measured how well phylogenetic informativeness predicts branch support on a node-by-node basis. To do so, genes were compared in pairs based on their predicted performance (DPI), comparing the predicted best with the predicted worst, the predicted second best with the predicted second worst, and so on. Then, we correlated their predicted proportionate performance:

(2) $$\begin{array}{c} \text{PPP}=\frac{{\text{DPI}}_{1}}{{\text{DPI}}_{1}+{\text{DPI}}_{2}}, \end{array}$$

where DPI_*i*_ > DPI_*j*_ and 0.5 ≤ PPP ≤ 1. With *n*_*i*_ denoting the number of nodes with higher support using gene *i*, and *n*_*j*_ denoting the number of nodes with higher support using gene *j*, the empirical proportionate performance of each gene pair in terms of nodes better supported was calculated as

(3) $$\begin{array}{c} \text{EPP}=\frac{n1}{n1+n2},\;\;\;\;\;\; \end{array}$$

where 0 ≤ EPP ≤ 1. To measure the strength of linear relationship between predicted and empirical performances, we calculated Pearson's correlation coefficient (*r*).

Third, we examined the cumulative global support for the well-established phylogeny as each gene was added according to several sampling schemes. To do so, we first calculated the proportionate likelihood-ratio support (PLRS) and the proportionate decay index support (PIDS) provided by each gene for each node in the well-established phylogeny. For each node, we divided the aLRT statistic and DI by the number of genes. Then, we calculated the average PLRS and PIDS across nodes for each gene. This average value can be interpreted as the relative contribution or global support of each gene for the well-established phylogeny. We plotted the cumulative path of the global support for each data set according to several sampling schemes. In an ideal experiment, this cumulative support would dramatically rise with the top-ranked prioritized loci and increase little as less informative markers were used. 

Sampling the genes from the highest to the lowest proportionate support would represent the ideal situation when deciding about sampling genes for sequencing. Logically, the other way around represents the worst-case scenario. We also plotted the hypothetical average path between these two extremes. Finally, we compared these paths with the plot when prioritizing sampling with DPI values. 

All alignment operations, data parsing, and communication of data to and from software were performed with Perl programming including Bioperl modules [38]. We also manipulated trees using Phyutility v2.2 [39]. All software used for the analyses mentioned corresponded to the Linux version. Only results from the ML analyses (i.e., ML-BP and aLRT) are shown. Relevant differences between ML and MP results are discussed in the text. 

## 3. Results 

### 3.1. Phylogenetic Informativeness Profiles

Graphical profiles of the phylogenetic informativeness for four loci scaled to match with the ultrametric trees (Figures 1–3) illustrated the great diversity of levels of informativeness among genes in all data sets. Plotted genes were chosen to provide extremal exemplars with different performances: the best and worst genes across the whole time scale and two other genes which showed most variation in recent compared with ancient times and vice versa. The phylogenetic informativeness profiles for the rest of the genes lie approximately within the range of the extremal profiles. The OrthoMaM data set illustrates this variation in informativeness well. Although the genes TP63 and LCA5 shared approximately the same number of sites (680 and 682 aa, resp.), LCA5 exhibited greater informativeness over the whole tree (Figure 1(b)). 

Compared to SYTL4 (2046 bp; Figure 1(c)), GFPT2 exhibited higher informativeness in recent times but lower informativeness for more ancient times and yet was composed of about the same number of sites (~2000 bp). The effects of variation of rates across sites on phylogenetic informativeness profiles were also observed in the simulated sequences (Figure 4). At the same global rate, the higher the gamma-shape parameter (alpha), the closer the profiles to a singular rate distribution. When alpha was low, corresponding to higher rate heterogeneity, the profiles peaked closer to recent times due to the presence of a set of faster evolving sites. 

Direct comparison between amino acid and DNA informativeness profiles for 40 genes for which amino acid and DNA were both extracted from OrthoMaM data sets demonstrated correlated patterns of informativeness, with two significant differences. First, amino acid profiles showed more variation from low to high informativeness potential. Second, DNA alignments had higher profiles than amino acid sequences (e.g., see SYTL4 Figures 1(b) and 1(c)), mainly due to their threefold greater number of sites. However, comparing per site profiles (data not shown), the differences in informativeness disappeared or in some cases even became inverted. Lower and flatter amino acid profiles were still present, probably due to silent substitutions. Silent substitutions, which mostly occur in the third position of a codon and have no effect upon the amino acid sequence, can cause a higher rate of evolution of nucleotide sites without affecting the rate of evolution of amino acid sites. Thus, silent substitutions in genes with flat amino acid profiles caused by the lack of sequence variation can produce higher DNA profiles. 

### 3.2. Phylogenetic Informativeness as Predictor of Node Identity and Branch Support

**Figure 5:**
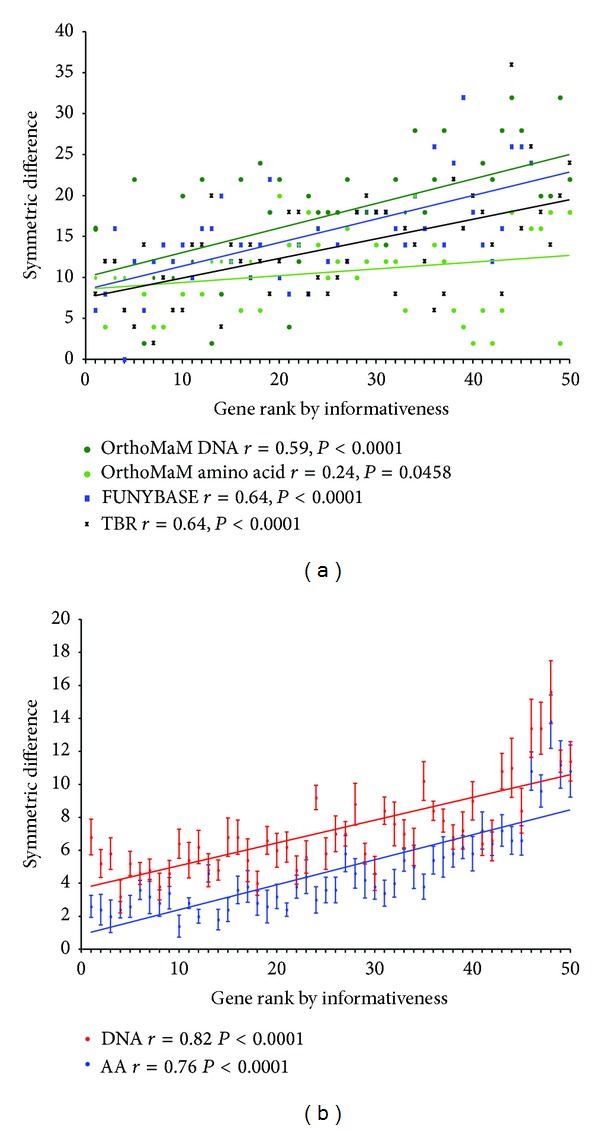
Phylogenetic accuracy. Symmetric-difference tree distance as a function of the gene ranking based on phylogenetic informativeness for (a) OrthoMaM amino acid (dark green circles), OrthoMaM DNA (light green circles), FUNYBASE (blue squares), and TBR (black crosses) data sets and (b) simulated amino acid (blue) and DNA (red) data. The symmetric difference was calculated comparing each gene tree estimated by ML to the well-established tree as obtained from the concatenated amino acid sequences. The corresponding Pearson's correlation coefficient (*r*) and *P* value for each data set are indicated. For the simulated data, the mean and its standard error were plotted using the replicates.

All DPI rankings significantly positively correlated with the ability to recover the correct topology (Figure 5). The OrthoMaM DNA data set exhibited the weakest (*r* = .24) and simulated amino acid and DNA data sets exhibited the strongest (*r* = .76 and *r* = .82, resp.) correlations. Other indices of informativeness such as gene length, number of variable sites, and number of parsimony informative sites were also tested for correlation with the symmetric differences (data not shown). All these other indices did not exhibit significant correlations except for the FUNYBASE and for the amino acid and DNA simulated data sets (all three correlations for these three data sets were significant, *P* < .05). The analyses using MP yielded strikingly similar results (data not shown). 

**Figure 6:**
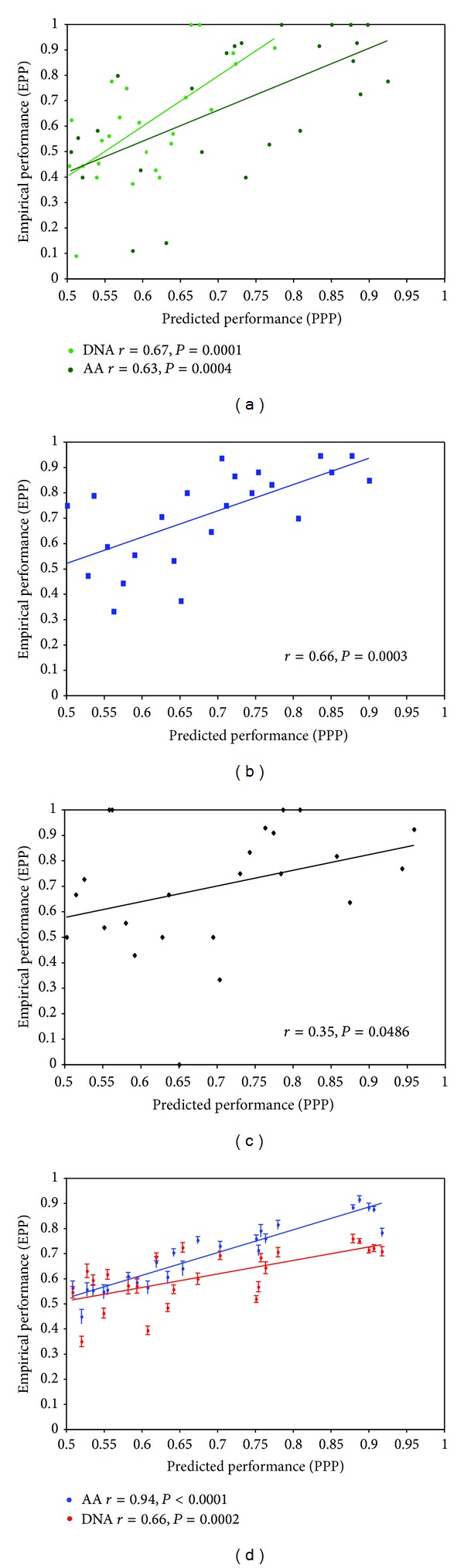
Phylogenetic informativeness as a predictor of maximum likelihood bootstraps (ML-BPs). Variation in ML-BP support (EPP) as a function of the variation in phylogenetic informativeness (PPP; see Material and Methods for more details) for (a) OrthoMaM amino acid (dark green), OrthoMaM DNA (light green), (b) FUNYBASE (blue), (c) TBR (black), and (d) simulated amino acid (blue) and DNA (red) data sets. The corresponding Pearson's correlation coefficient (*r*) and *P*value for each data set are indicated. For the simulated data, the mean and its standard error were plotted using the replicates.

Phylogenetic informativeness as predictor of ML-BP yielded significant correlations for all data sets (Figure 6). The TBR data set exhibited the weakest (*r* = .34) and the simulated amino acid alignments the strongest (*r* = .94) correlation. Generally, stronger correlations with accuracy and robustness (Figures 5 and 6) were revealed in the simulated data sets than in the empirical data. Other summary statistics for genes such as gene length, number of variable sites, and number of parsimony informative sites also correlated with performance. Locus length was significantly correlated only with ML-BP in the FUNYBASE data set (*n* = 23, *r* = .65, *P* < .001). In addition, in all data sets with the exception of the DNA OrthoMaM data set, ML-BP exhibited significant correlations (*P* < .05) with the number of variable and parsimony informative sites. For the DNA OrthoMaM data set, the only measure that was significantly correlated with node identity and branch support was the phylogenetic informativeness. To ensure that this result did not arise as a peculiar consequence of our length-based selection of genes, we evaluated these correlations with an additional, nonoverlapping subset of OrthoMaM DNA sequences with a lower mean number of sites (~1000 bp). The phylogenetic informativeness was again the only measure significantly correlated to both node identity (*n* = 50, *r* = .44, *P* < .001) and branch support (*n* = 25, *r* = .59, *P* < .001). MP analyses yielded similar results. For MP, the DNA simulated alignments exhibited the weakest (*n* = 25, *r* = .44, *P* = .013) and the FUNYBASE the strongest (*n* = 25, *r* = .79, *P* < .0001) correlations. 

**Figure 7:**
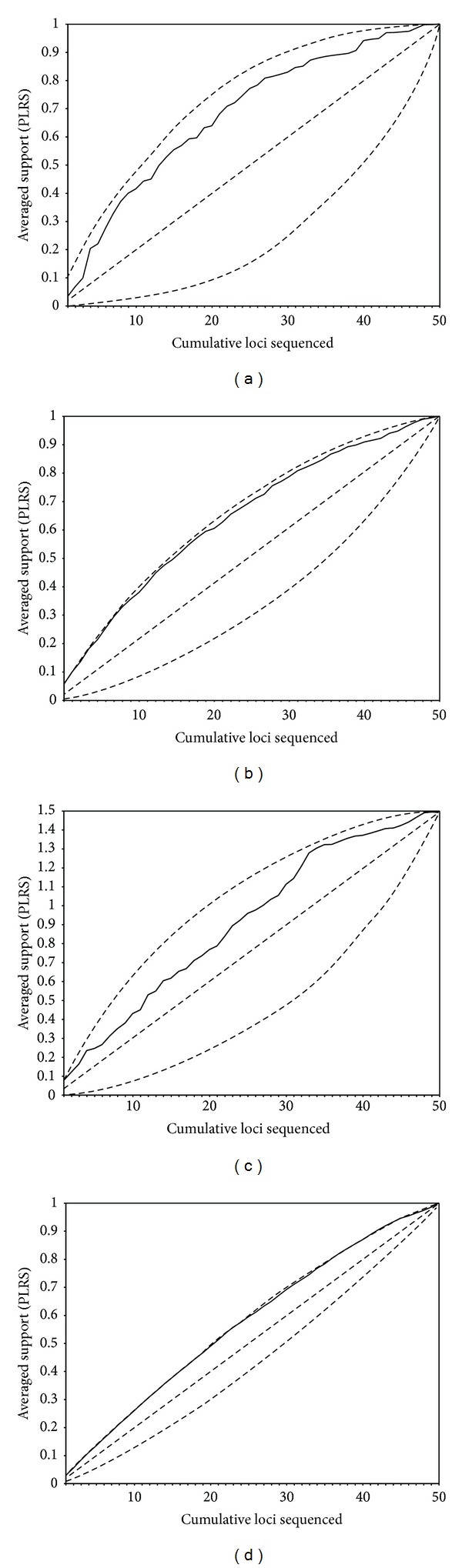
Cumulative proportionate likelihood-ratio supports (PLRS) averaged across nodes for (a) OrthoMaM amino acid, (b) FUNYBASE, (c) TBR, and (d) simulated amino acid data sets. Genes are ranked by differential phylogenetic informativeness encompassing all branches in the tree. The upper dashed line represents cumulative PLRS when loci are prioritized, posthoc, from highest to lowest PLRS values. The lower dashed line represents cumulative PLRS when loci are prioritized, posthoc, from lowest to highest PLRS values. The intermediate dashed line is the hypothetical average one would achieve sampling at random from loci available.

**Figure 8:**
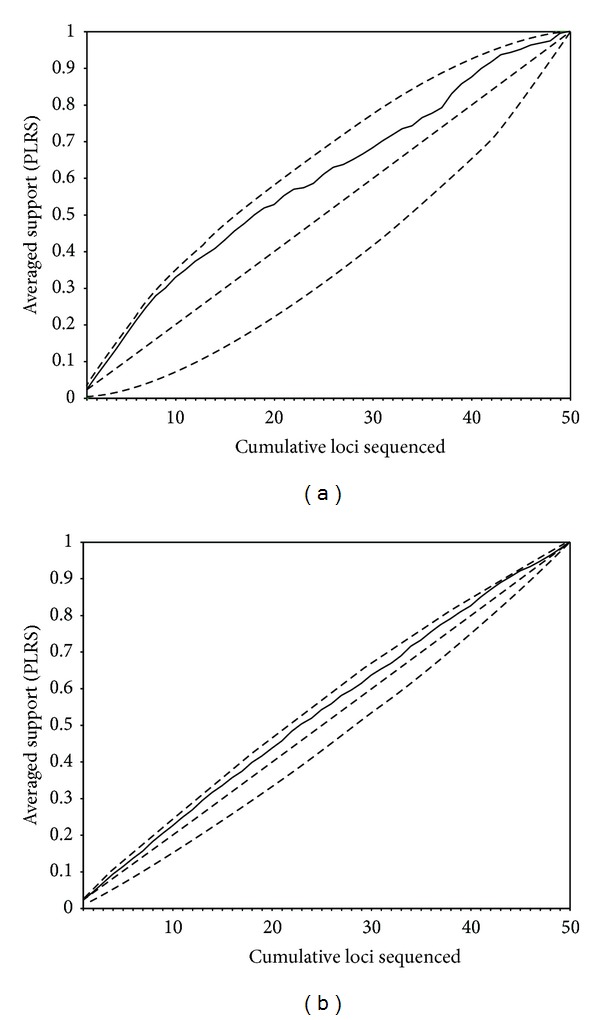
Cumulative proportionate likelihood-ratio supports (PLRS) averaged across nodes for (a) OrthoMaM DNA and (b) simulated DNA data sets. Genes are ranked by differential phylogenetic informativeness encompassing all branches in the tree. The upper dashed line represents cumulative PLRS when loci are prioritized, posthoc, from highest to lowest PLRS values. The lower dashed line represents cumulative PLRS when loci are prioritized, posthoc, from lowest to highest PLRS values. The intermediate dashed line is the hypothetical average one would achieve sampling at random from loci available.

DPI rankings provided close to the optimal experimental design path (i.e., sampling the genes from the highest to the lowest average PLRS, which would represent the ideal situation when deciding about sampling genes for sequencing) for all amino acid data sets (Figure 7), outperforming haphazard sampling, especially for FUNYBASE and simulated amino acid data. DPI rankings for the TBR data set showed more deviation from the ideal sampling. Prioritizing with DPI rankings for both OrthoMaM and simulated DNA data (Figure 8) also yielded results close to optimal experimental design path, outperforming the haphazard path. The best and worst experimental design paths for DNA data sets showed less difference from each other than did their respective amino acid counterparts. This result is consistent with the highest variability in amino acid informativeness potential mentioned earlier when comparing overlapping genes for amino acid and DNA OrthoMaM data sets. A similar trend was observed in both simulated and empirical data. Empirical data showed higher variability in the performance of individual genes. We also repeated cumulative plots ranking genes based on the other summary statistics mentioned previously: gene length, number of variable sites, and number of parsimony informative sites. For all data sets with the exception of DNA OrthoMaM, these rankings performed better than haphazard sampling. For the DNA OrthoMaM data set, the phylogenetic informativeness ranking performed noticeably better than these other measures, which in some parts of the plot crossed or were below the average path. The analyses using MP yielded similar results. 

## 4. Discussion 

We systematically examined the Townsend [5] phylogenetic informativeness as a metric for assessing phylogenetic signal. Our results demonstrate that prioritizing rankings obtained with phylogenetic informativeness was significantly correlated with the ability of a gene to recover the right topology as well as with higher branch support measures. In addition, we found that the informativeness metrics significantly outperformed haphazard experimental design and predicted a close-to-optimal prioritization of gene sequencing. Although Townsend [5] phylogenetic informativeness was based on analysis of the canonical four-taxon problem and although it does not specifically account for the misleading effects of homoplasy, our analysis suggests that the metric is robust despite these limitations. We examined its predictions in phylogenomic data sets spanning diverse time scales and taxonomic groups for both amino acid and DNA sequences and supplemented our empirical analyses with controlled simulations. Furthermore, we validated the results for both parsimony and maximum likelihood optimality criteria. We conclude that phylogenetic informativeness profiles provide advantageous guidance for phylogenetic projects in the selection and prioritization of loci to sequence for maximal return on effort and investment. 

Despite its crucial role, pursuit of analytical methods for experimental design in phylogenetics has been sparse. Until recently, the only prominent procedure developed to deal with the question of experimental design in the context of topological uncertainty has been the empirical saturation plot [40]. In this plot, a lack of more or less increasing linear sequence divergence with time would indicate saturation in the set of characters analyzed. However, the plots are hard to fit unambiguously to data and do not lend themselves to immediate quantifications of informativeness for specific epochs. Other graphical methods to visualize phylogenetic signal have been advanced, such as likelihood mapping [41] and plotting Treeness triangles [42]. Although well suited for post hoc analyses, a major issue with these graphical approaches is that they are not easy to interpret or very practical for large-scale surveys. More importantly, neither puts forward an applied methodology for ranking genes for phylogenetic utility. Recently, responding to this necessity, two nongraphical strategies were proposed: (i) [43] suggests ranking genes by comparing the cophenetic correlation coefficients among individual protein distances matrices and (ii) [16] advocates ranking phylogenetic performance of genes using a topological metric, comparing individual gene topology against a reliable reference tree. These two approaches also provide insights into conflicting phylogenetic signal among genes, a practice followed more or less formally by phylogeneticists [44]. Both methods were tested in a similar set of fungal genomes, however, yielding different gene rankings [16]. Apparently, using topological distances is a superior strategy [16]. Although they have utility for ranking genes, such topological distance measures require a reference topology for the taxa of interest and extensive individual gene phylogenetic analyses. Since they yield an absolute rank rather than a function that modulates over historical time, they do not provide a domain of utility that may be extended to taxa within or outside the original analysis and thus cannot be the focus to determine genes that will be most useful for investigating phylogenetic questions at a given taxonomic level. 

In order to estimate phylogenetic informativeness, one requires the site rate distribution for each locus. To obtain the rates, two prior pieces of information are needed: (1) an alignment of loci of interest pruned to contain a set of taxa for which the tree topology is fairly well known and (2) an ultrametric tree for those taxa. The ultrametric tree can be either a chronogram—an ultrametric tree with branch lengths proportional to time—or it can be in unspecified molecular evolutionary units. This prior information may be derived from three potential sources: (1) preliminary data on the candidate loci from a well-studied subset of the taxa of interest; (2) data on the candidate loci from a well-studied sister clade; or (3) comparative genomic data from sequenced genomes within and/or outside the clade of interest. Informativeness profiles can be generated with the online application PhyDesign [27] and used to rank loci based on their ability to resolve nodes of interest, allowing assessment of the relative signal of genes within large data sets. However, erroneous rate estimations, such as those caused by an incorrect input topology, will affect the accuracy of informativeness profiles. Thus, iterative refinement/recalculation of PI while increasing taxonomic sampling is recommended for researchers seeking to identify the best candidate loci for phylogenetic reconstruction.

To apply all the information at hand and to assess results in a familiar way in this study, we used the phylogenetic informativeness of genes over the full epoch integrating from time 0 to the root. While generality and ease of presentation were gained by this procedure, the strength of the correlations observed herein was likely reduced as a cost of that generality. In fact, the rank order of genes by informativeness varies over history due to the pattern of variation of rates among sites. Comparison of the profiles of informativeness for the different data sets against their chronograms (Figures 1–3) illustrated the different gene potential for signal across their evolution. As a simple example, in Figure 2, MS409 (MetRS, mitochondrial methionyl-tRNA synthetase) from FUNYBASE shows a great potential for questions of recent fungi evolution and much lower potential signal for all of the rest of fungal history. In addition, genes showing the same mean rate can have different phylogenetic profiles [5], even though it is a common practice to talk about slow-evolving genes and rapid-evolving genes to define their temporal applicability. An incontrovertible example of this is found in the simulated sequences profiles (Figure 4), in that each variant with a different gamma-shape parameter showed a different informativeness profile. Numerous examples of the importance of incorporating rate variation among sites for the correct phylogenetic inference are found in the literature [45–48]. 

Phylogenetic informativeness ably predicted gene performance in all data sets, encompassing diverse evolutionary contexts. In contrast, it is common practice to explore the adequacy of a methodology for a single set of empirical data or under a single criterion for phylogenetic inference [5, 16, 43]. However, due to the complexity of the biological process that generates phylogenetic data, extrapolating strong conclusions from individual data sets can be inadvisable. 

Initially, we had expected that phylogenetic noise might hinder gene prediction of performance for data sets with more ancient nodes. Some theoretical work associated with particular data sets has indicated that homoplasy obscures the phylogenetic signal for periods older than 600 MA, and eliminates the signal as 1000 MA is approached [42, 49]. However, we did not observe a pattern indicating that phylogenetic informativeness predicts better for one data set or another based on the age of the events encompassed by their phylogenies.

The contrast between the hypothetical ideal and worst prioritizing rankings for DNA data sets (Figure 8) was less than the contrast between their respective amino acid counterparts (Figure 7). A greater similarity in phylogenetic performance among DNA sequences than among amino acid sequences might be responsible for this pattern. The greater similarity of informativeness for DNA sequences can be attributed to the consistent presence of a homogeneous class of fast-evolving silent sites in DNA sequences. In contrast, amino acid sequences have no consistent, a priori identifiable fast-evolving site class like degenerate third codon position sites in DNA but instead may range from extreme constraint on all sites to lack of constraint on many sites. Thus, polymorphism at silent coding sites can lead to high-informativeness DNA profiles for genes with flat amino acid profiles and, for the same reason, make DNA profiles more similar to each other compared with amino acid profiles. The number of characters and the signal retention for each character will dictate the different phylogenetic performance of these two data types [50]. For most inference purposes, the net phylogenetic informativeness is the prediction of interest, as it should correlate with empirical results, such as the degree of support of a node. However, per site phylogenetic informativeness can be calculated to quantify the cost versus benefit of sequencing and to compare relative phylogenetic potential without the confounding effect of sequence length. For example, a top-ranked gene may show good net phylogenetic informativeness profiles, but there may be one or more shorter markers (requiring less sequencing effort) that may exhibit better per site profiles. A combination of shorter genes requiring the equivalent sequencing effort of a longer marker might lead to the best results. 

Two explanations may underlie the observation that amino acid data sets tended to show stronger correlations between informativeness rankings and tree distances or BPs. The expanded character-state space accessible for amino acids compared with DNA sequences can diminish the potential misleading effects of homoplasy in phylogenetic inference. Because signal is accounted for in the Townsend [5] informativeness but the potential for misleading homoplasy is not, greater homoplasy in DNA sequences might have led to worse predictions for DNA markers than for amino acid markers. Although functional constraints in proteins can limit character-state space available for a given amino acid site, simulations indicate that small increases in the character-state space increase accuracy of phylogenetic inference [50]. A second reason for the better performance of informativeness predictions for amino acid sequences could be better alignment. The long evolutionary distances between sequences used can make alignment of homologous residues of DNA sequence much more challenging than alignment of corresponding amino acid sequences. 

Simulation studies have been successfully applied to address questions of character and taxon sampling strategies [51–53] and also for comparing methods of branch support [54, 55]. Differences between results achieved with empirical data sets and results achieved with simulated data sets are likely to derive from the strict adherence to the model of evolution in simulations compared to frequent deviation from the model typical in empirical data. Simulations oversimplify the substitution process. To perform simulations, we incorporated a specific evolutionary model. Thus, the regularities of the model dictated regularity in the results. Stochasticity in the nature of the substitution process in empirical data precludes better predictions of gene performance in empirical data sets than in simulated data sets. Even when phylogenetic informativeness predicts that there are a considerable number of sites evolving at optimal rates, changes will not necessarily map at all or in sufficient numbers to the branches of interest. Mutation, selection, and genetic drift processes determine the number and position of differences observed. Stochasticity of the substitution process will affect any attempt at gene performance prediction. 

We found that gene length, number of parsimony informative sites, and/or number of variable sites were also significantly correlated with the tree distances measures for the FUNYBASE and the simulated data sets. For all data sets except OrthoMaM, the number of variable sites and the number of parsimony informativeness sites were also significantly correlated with BPs. For genes in FUNYBASE, significant correlations of gene length and number of variable sites with gene performance have been observed previously [16]. Accordingly, [55] found that these three indices were significantly correlated with bootstrap values for some branches. However, none of these parameters could systematically be used as a predictor of single gene performance [55]. Moreover, the number of variable sites and particularly parsimony informative sites represent posthoc indices giving estimates of the amount of signal present in the alignments that cannot be justifiably projected to a different set of taxa or to a novel depth in a phylogeny, limiting their utility. Interestingly, the only parameter that predicted the gene performance in OrthoMaM DNA data set was the phylogenetic informativeness metric. This result reinforces the idea that incorporating character rate evolution for gene performance predictions is a key factor and that phylogenetic informativeness metrics can be successfully applied to systematically facilitate more cost-effective phylogenetic research. Extending the method of Townsend [5] to account for additional nuances of molecular evolution, such as the accumulation of homoplasy [56], will further bolster its applications to phylogenetic experimental design.

## 5. Conclusions

Phylogenetic analyses with broad taxonomic sampling, such as Tree of Life projects, can, with just a few of the right genes, accumulate sufficient data to build a reliable phylogeny (e.g., less than 10 genes) [16]. Ideally, the set of genes required would be minimized. Thus, as a consequence of the extensive selection of potential loci that may be pursued based on genome projects, a decision on what genes need to be sequences has to be made. Despite this need, pursuit of analytical methods for experimental design in phylogenetics has been sparse. We explored the impact of using the Townsend [5] phylogenetic informativeness and found it to be an advantageous procedure for using genome-scaled sequence data to identify loci with high utility for phylogenetic inference. This choice of genes is critical, not only for their global performance across depths of the tree but also for their performance in resolving particular timescales. By estimating the full distribution of rates at each site, phylogenetic informativeness profiles showed how signal content varies among genes and across time. Thus, one may select genes that will perform best for the epoch of interest, whether for recent divergence times or for more ancient divergence times. Prioritization predictions made by the Townsend [5] phylogenetic informativeness correlate with the accuracy and robustness with which a gene sequence recovers the correct topology. These predictions are valid for amino acid and DNA markers of diverse groups of organisms spanning broad time scales, especially when the time scale is not significantly deeper than the peak of informativeness. This quantitative and objective informativeness metric can play a critical role in augmenting the efficiency and accuracy of many phylogenetic studies at multiple time scales. 

## Supplementary Material

The supplementary material contains spreadsheets listing the genes for the empirical datasets used in the manuscript: FUNYBASE, TBR, DNA OrthoMaM, and AA OrthoMaM. Genes were ranked by the differential phylogenetic informativeness (DPI). DPI of each gene was evaluated quantitatively by integrating over the phylogenetic informativeness profile from the leaves to the root of the tree. Thus, DPI encompassed all branching points of the phylogenies, providing a summary of the relative informativeness of each gene to resolve all nodes in the phylogeny.

## Figures and Tables

**Table 1:** Data sets used in the study.

Data set	Source	Gene number	Mean length ± SD
OrthoMaM (AA)	Ranwez et al. (2007) [12]	50	668.8 ± 13.3
OrthoMaM (DNA)	Ranwez et al. (2007) [12]	50	2013.0 ± 43.5
FUNYBASE (AA)	Marthey et al. (2008) [13]	46	284.4 ± 130.0
TBR (AA)	Taylor and Berbee (2006) [15]	50	241.3 ± 106.3
Simulations (AA)	Seq-Gen	50	300 ± 0
Simulations (DNA)	Seq-Gen	50	300 ± 0
